# Chronic oral or intraarticular administration of docosahexaenoic acid reduces nociception and knee edema and improves functional outcomes in a mouse model of Complete Freund’s Adjuvant–induced knee arthritis

**DOI:** 10.1186/ar4502

**Published:** 2014-03-10

**Authors:** Ana M Torres-Guzman, Carlos E Morado-Urbina, Perla A Alvarado-Vazquez, Rosa I Acosta-Gonzalez, Aracely E Chávez-Piña, Rosa M Montiel-Ruiz, Juan M Jimenez-Andrade

**Affiliations:** 1 Unidad Académica Multidisciplinaria Reynosa Aztlán, Universidad Autónoma de Tamaulipas, Calle 16 y Lago de Chapala, Col Aztlán, CP 88740 Reynosa, Tamaulipas, México; 2 Laboratorio de Farmacología, Programa Institucional de Biomedicina Molecular, Escuela Nacional de Medicina y Homeopatía del Instituto Politécnico Nacional, México, DF, México

## Abstract

**Introduction:**

Clinical and preclinical studies have shown that supplementation with ω-3 polyunsaturated fatty acids (ω-3 PUFAs) reduce joint destruction and inflammation present in rheumatoid arthritis (RA). However, the effects of individual ω-3 PUFAs on chronic arthritic pain have not been evaluated to date. Thus, our aim in this study was to examine whether purified docosahexaenoic acid (DHA, an ω-3 PUFA) reduces spontaneous pain-related behavior and knee edema and improves functional outcomes in a mouse model of knee arthritis.

**Methods:**

Unilateral arthritis was induced by multiple injections of Complete Freund’s Adjuvant (CFA) into the right knee joints of male ICR adult mice. Mice that received CFA injections were then chronically treated from day 15 until day 25 post–initial CFA injection with oral DHA (10, 30 and 100 mg/kg daily) or intraarticular DHA (25 and 50 μg/joint twice weekly). Spontaneous flinching of the injected extremity (considered as spontaneous pain-related behavior), vertical rearing and horizontal exploratory activity (considered as functional outcomes) and knee edema were assessed. To determine whether an endogenous opioid mechanism was involved in the therapeutic effect of DHA, naloxone (NLX, an opioid receptor antagonist, 3 mg/kg subcutaneously) was administered in arthritic mice chronically treated with DHA (30 mg/kg by mouth) at day 25 post–CFA injection.

**Results:**

The intraarticular CFA injections resulted in increasing spontaneous flinching and knee edema of the ipsilateral extremity as well as worsening functional outcomes as time progressed. Chronic administration of DHA, given either orally or intraarticularly, significantly improved horizontal exploratory activity and reduced flinching behavior and knee edema in a dose-dependent manner. Administration of NLX did not reverse the antinociceptive effect of DHA.

**Conclusions:**

To the best of our knowledge, this report is the first to demonstrate DHA’s antinociceptive and anti-inflammatory effects as individual ω-3 PUFAs following sustained systemic and intraarticular administration in a mouse model of CFA-induced knee arthritis. The results suggest that DHA treatment may offer a new therapeutic approach to alleviate inflammation as well as a beneficial effect on pain-related functional disabilities in RA patients.

## Introduction

Rheumatoid arthritis (RA) is one of the most common forms of chronic inflammatory arthritis and has a significant negative impact on society [1-3]. Joint pain is the most frequent symptom in patients with RA and generally decreases their functional status and quality of life, as it is strongly associated with physical disability, decreased mobility, depression, sleep disturbances and increased health-care costs [4-7]. Current therapies to alleviate joint pain have limited efficacy and/or induce several side effects, given that patients with RA may be exposed to long-term use of these analgesics [8-11]. Therefore, there is a clinical need to develop mechanism-based therapies with better efficacy and fewer side effects than those currently used to treat joint pain in patients with RA.

Clinical reports have shown that intake of ω-3 polyunsaturated fatty acids (ω-3 PUFAs) is associated with a reduction in the severity of RA [12-14]. In the majority of these studies, however, a heterogeneous mixture of the two main active ω-3 PUFAs has been used: eicosapentaenoic acid (EPA) and docosahexaenoic acid (DHA) [15,16]. Additionally, investigators in several studies have shown that EPA and DHA have remarkably different biological effects. For example, whereas EPA enhances osteoclastogenesis, DHA is a potent inhibitor of osteoclastogenesis [17,18]. Moreover, DHA, but not EPA, reduces the mouse ear edema induced by phorbol ester [19]. Thus, studies examining the effects of pure ω-3 PUFAs on arthritis pathology and arthritis-induced pain are needed.

Currently, it is believed that spontaneous arthritic pain (that is, joint pain at rest) [20,21] and movement-evoked pain are driven primarily by a chronic joint inflammation that induces both peripheral and central sensitization [22]. Recently, researchers in preclinical studies have reported that osteoclasts (that is, the cells that resorb bone) play a significant role in arthritis-induced bone loss and contribute to the etiology of arthritic pain [23,24]. As inflammation and osteoclastogenesis have been shown to be reduced by DHA [17,18,25,26] in different animal models, and because these processes have also been demonstrated to occur in RA [24,27], we sought to evaluate the effect of chronic administration of pure DHA on knee edema, spontaneous nociception and pain-related functional disabilities in a mouse model of knee arthritis induced by Complete Freund’s Adjuvant (CFA).

Intraarticular delivery has been widely used to administer different drugs to treat symptoms associated with arthritis (for example, pain and inflammation) because this method, as compared to systemic administration, may allow delivery of greater concentrations, increased time that the drug is localized within the joint and fewer side effects [28-31]. Although the effects of DHA have previously been tested in acute pain models after oral [32], intrathecal [33,34] and intraplantar [35,36] injection, no data regarding the efficacy of DHA in arthritic pain models following intraarticular administration have been published to date. Thus, the second major goal of this study was to determine whether sustained intraarticular DHA administration reduces spontaneous pain-related behaviors and knee edema and improves functional outcomes in a mouse model of arthritis of the knee joint.

## Methods

### Animals

Experiments were performed on a total of 83 male adult ICR mice (Harlan Laboratories, Mexico City, Mexico) weighing 18 to 25 g at the beginning of the experiments. All animals were housed in our facilities at a constant temperature (22°C to 24°C) and had free access to food and drinking water before the experiments. This study was approved by the Comité de Ética Institucional de la Unidad Académica Multidisciplinaria Reynosa Aztlán de la Universidad Autónoma de Tamaulipas (CEI-UAMRA-2012-10). All experiments were conducted in accordance with the Ethical Guidelines for Investigations of Experimental Pain in Conscious Animals [37]. At the end of the experiments, all animals were killed in a CO_2_ chamber.

### Drugs

CFA (catalog no. F5881), DHA (catalog no. D2534), olive oil (vehicle; catalog no. W530191) and naloxone (NLX; catalog no. N7758) were purchased from Sigma-Aldrich (Toluca, Mexico). DHA was dissolved in olive oil and administered by oral gavage or intraarticular injection.

### Complete Freund’s Adjuvant injection

A version of a previously validated model of arthritic inflammation of the knee joint [38-40] was produced by performing four intraarticular injections of CFA (10 μl) at days 0, 7, 14 and 21 unilaterally into the right knee joint. Briefly, mice were anesthetized using a mixture of ketamine/xylazine (100 mg/kg and 5 mg/kg by intraperitoneal injection, respectively), followed by an intraarticular injection of CFA using a 30-gauge, 0.5-inch needle that was fitted with cannulation tubing such that only 2 to 3 mm of the needle was allowed to puncture the joint. CFA was injected through the patellar ligament into the articular space using the femoral condyles as a guide.

### Behavioral measures of arthritic joint pain

Behavioral measures of arthritic joint pain included flinching of the affected limb (spontaneous pain), number of total vertical rearings requiring the use of both hind limbs and horizontal exploratory activity (used as functional outcomes of the affected limb). Briefly, mice were placed in open cylindrical Plexiglas observation chambers (height 30 cm and inside diameter 20 cm) for 20 minutes to allow the mice to acclimate to their surroundings. Mirrors were placed behind the chambers to enable unhindered observation. The number of spontaneous flinches was recorded during a 5-minute observation period as previously described [41]. Flinches were defined as the number of times the animal raised its hind paw, which was used as a measure of spontaneous arthritic pain. Although spontaneous flinching is not present in patients with RA, this behavior may reflect the spontaneous pain that arthritic patients experience without movement (that is, pain at rest) of the affected extremity [21,41,42].

The number of total vertical rearings was recorded during a 5-minute observation period while the animals were in the open Plexiglas observation chamber. Total vertical rearings was defined as the number of times the animals stood on both hind limbs while supporting their entire body weight [43]. A reduction in vertical rearing in mice is consistent with the reduction in movement or joint-loading observed in RA patients [21,42].

Once flinching behavior and vertical rearing had been evaluated, horizontal exploratory activity was quantified using a modified open-field paradigm. Mice were placed in the center of a rectangular acrylic chamber (18 × 27 × 15 cm). The chamber was divided into a grid of equally sized rectangles (nine in total measuring 9 × 6 cm each) using lines drawn on the chamber floor for visual quantification. The total number of times each line was crossed in a 2-minute period was quantified by the experimenter [44,45].

### Study design

#### Chronic oral administration of docosahexaenoic acid

After an acclimatization period of 1 week in our facilities, mice were randomly divided into four groups (*n* = 7 to 9 per group): one group comprising mice that received oral injections containing CFA and olive oil (vehicle) and three groups comprising mice that received oral injections of CFA and DHA concentrations of 10, 30 or 100 mg/kg. DHA or vehicle was given 2 hours before behavioral analysis on a daily basis from day 15 until day 25 after first CFA injection. Behavioral measures of arthritic joint pain were evaluated at days 0, 11, 18 and 25 after the initial CFA injection. The oral doses of DHA given in this study were similar to those used in a previous study in mice in which the researchers demonstrated a significant reduction in glucose and triglyceride levels [46]. The toxicological profile of DHA demonstrates that this oil is safe in rats (up to a consumption level of 3,290 mg/kg/d) over 90 days of toxicological evaluation [47,48].

#### Chronic intraarticular administration of docosahexaenoic acid

To determine the effect of chronic intraarticular administration of DHA, mice were randomly divided into three groups (*n* = 9 or 10 per group): mice that received intraarticular injections of CFA and olive oil (vehicle) and mice that received intraarticular injections of CFA and DHA (25 and 50 μg/joint). Five microliters of DHA or vehicle were injected into the joint space of the anesthetized animal as described above. DHA was administered at days 15, 19 and 22 after the initial CFA injection. For this protocol, DHA was administered 3 days before behavioral analysis to avoid interference of intraarticular administration of CFA. Behavioral measures of arthritic joint pain were evaluated at days 0, 15, 18 and 25 after the initial CFA injection. At day 15, behavioral measures were performed immediately prior to the intraarticular injection of DHA to prevent any interference with behavioral analysis by the puncture itself. The intraarticular doses of DHA employed were based on those described in previous studies utilizing intrathecal or intraplantar delivery of DHA in acute pain models [33,36].

To determine whether oral or intraarticular administration of DHA by itself modified behavioral measurements, treatment-naïve mice received oral 30 mg/kg DHA once daily for 10 days or intraarticular 50 μg/joint DHA at days 1, 4 and 7. Next, the number of flinches, vertical rearings and horizontal exploratory activity were evaluated at days 0, 1, 4, 8 and 11 after DHA administration.

#### Acute oral administration of docosahexaenoic acid

Several studies have shown the effect of DHA in different acute pain models [32,33]. To determine the acute effect of oral administration of DHA on pain-related behaviors in a mouse model of arthritis, we performed behavioral analysis at 1, 2 and 24 hours after a single oral administration of 30 mg/kg DHA at day 15 after the initial CFA injection. The maximum antinociceptive effects were observed between 30 and 100 mg/kg DHA, so only the 30 mg/kg dose was evaluated further in acute administration studies.

#### Involvement of opioid mechanism in antinociceptive effect of docosahexaenoic acid

To evaluate the involvement of an endogenous opioid pathway, at day 25 after CFA injection, mice with CFA-induced arthritis were randomly divided into three groups of six animals each: (1) mice that received injections of CFA and olive oil orally (vehicle) as well as subcutaneous injections of 3 mg/kg NLX, (2) mice that received injections of CFA and 30 mg/kg DHA orally as well as 3 mg/kg NLX subcutaneously and (3) mice that received CFA injections and 30 mg/kg DHA orally as well as distilled water subcutaneously (vehicle). DHA was given daily from day 15 until day 25 after the initial CFA injection. At day 25, 90 minutes after DHA administration, NLX or distilled water was subcutaneously administered. Next, behavioral analysis was performed at 30 minutes after NLX administration to ensure that mice were tested within the therapeutic time window of the antagonist [49,50].

#### Determination of knee diameter

To quantitatively determine the anti-inflammatory effect of DHA after oral or intraarticular administration, the diameter of the knee joint just below the level of the patella was measured in the anesthetized animal using a digital caliper. In animals that received oral DHA, the knee joint diameter was measured on day 0 (before the initial CFA injection) and on days 14, 18 and 25 after the initial injection of CFA. When DHA was administered intraarticularly, the knee joint diameter was measured on day 0 (before the CFA injection) and on days 15, 18 and 25 after the initial CFA injection.

To determine the effect of a single oral administration of DHA on knee edema in mice with CFA-induced arthritis, the knee diameter was measured at 2 and 24 hours after oral administration of DHA (30 mg/kg) at day 15 after the initial CFA injection. To determine whether intraarticular administration of DHA by itself modified knee joint diameter, knee joints were evaluated in treatment-naïve mice on days 0, 4, 8 and 11 following initial intraarticular DHA administration. The results are expressed as percentage of inflammation compared to that in the contralateral knee joint: Inflammation (%) = [(Ipsilateral knee diameter - Contralateral knee diameter)] ÷ Contralateral knee diameter.

### Data analysis

A two-way repeated-measures analysis of variance (ANOVA) followed by Bonferroni *post hoc* correction was used to compare the behavioral measures between the experimental groups at different time points. The magnitude of the anti-inflammatory effect of DHA was determined using two-way ANOVA followed by the Bonferroni *post hoc* correction. The statistical significance level was set at *P* < 0.05. In all possible cases, the investigator responsible for behavioral testing, measuring and counting was blinded to the experimental conditions of each animal.

## Results

### Effect of chronic oral administration of docosahexaenoic acid on pain-related behaviors

Mice that received only an intraarticular injection of saline or DHA showed minimal spontaneous nociceptive behavior or impairment of functional outcomes (data not shown). In contrast, mice that received CFA injections exhibited a greater number of flinches (considered spontaneous pain-related behavior) than observed in sham-treated mice from day 4 through day 25 after the initial CFA injection (data not shown). Additionally, the number of line crosses in the open field test (pain-related functional disability) and vertical rearings (pain-related functional disability) were significantly reduced in mice that received CFA injections than sham-treated mice as time progressed (data not shown).

**Figure 1:**
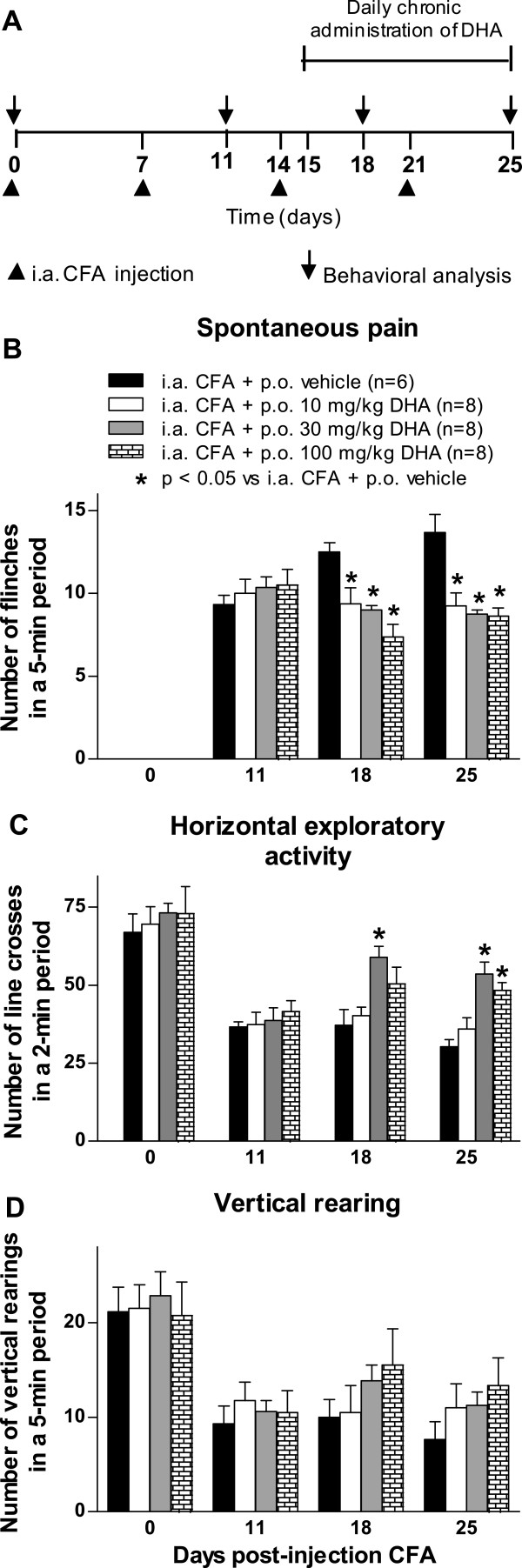
**Chronic oral administration of docosahexaenoic acid reduces pain-related behaviors and functional disability in arthritis induced by Complete Freund’s Adjuvant.** Docosahexaenoic acid (DHA) or vehicle was orally administered (p.o.) from day 15 until day 25 after initial Complete Freund’s Adjuvant (CFA) injection. **(A)** Assessment of arthritic joint pain included flinching of the affected limb (spontaneous pain-related behavior), horizontal exploratory activity and vertical rearing (pain-related functional disability), which were measured at days 0, 11, 18 and 25 following initial CFA injection. The intraarticular (i.a.) CFA injections resulted in significantly increasing spontaneous flinching and significantly decreasing functional outcomes as time progressed. **(B)** Three doses of DHA significantly reduced the number of spontaneous flinches in a dose-dependent manner; however, the maximum effect on spontaneous flinching was observed at a dose of 30 mg/kg. **(C)** Horizontal exploratory activity increased significantly in mice that received CFA injections following DHA treatment at doses of 30 and 100 mg/kg. **(D)** Oral administration of DHA did not significantly increase the number of vertical rearings at the three doses evaluated. Each bar represents the mean ± SEM. *P < 0.05 vs i.a. CFA + p.o. vehicle compared to its respective time period by two-way repeated-measures analysis of variance followed by *post hoc* Bonferroni correction.

To determine the effect of chronic oral administration of DHA on spontaneous nociceptive behavior and on pain-related functional disabilities, DHA was administered daily from day 15 until day 25 after the initial CFA injection (Figure 1A). Mice with CFA-induced arthritis and treated with DHA at three different doses (10, 30 and 100 mg/kg by oral administration) showed significantly fewer flinches than mice with CFA-induced arthritis and treated with vehicle (Figure 1B). The antinociceptive effect of DHA was maintained until day 25. Additionally, chronic oral administration of DHA (at 30 and 100 mg/kg) significantly reversed the CFA-induced impairment of horizontal exploratory activity. This beneficial effect was also maintained until day 25 after the initial CFA injection (Figure 1C). The antinociceptive effect of DHA on these two pain-related behaviors reached a maximum effect between the doses of 30 and 100 mg/kg. DHA administration did not significantly change the CFA-induced reduction in vertical rearing (Figure 1D).

### Effect of chronic oral administration of docosahexaenoic acid on knee edema

**Figure 2:**
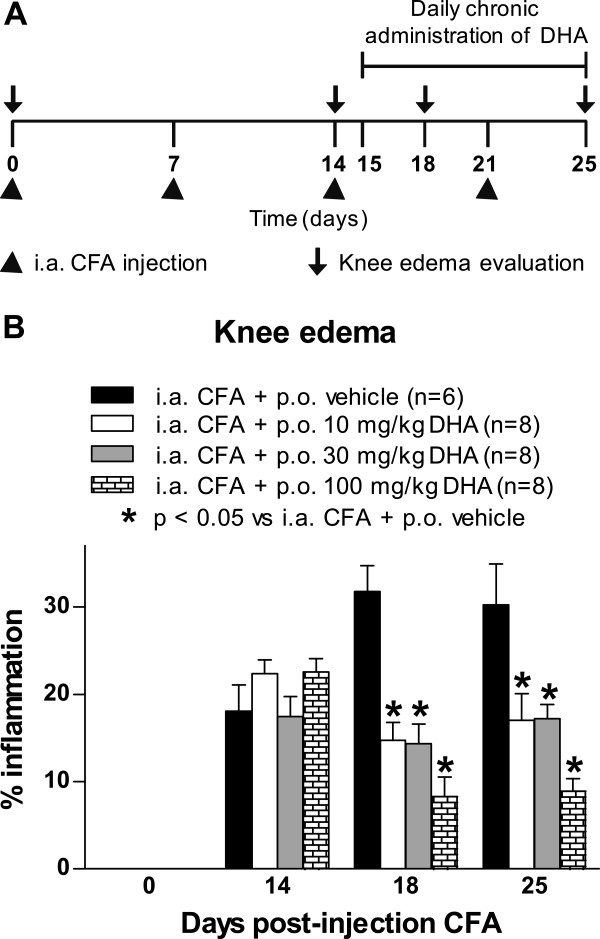
**Chronic oral administration of docosahexaenoic acid reduces knee edema in arthritis induced by Complete Freund’s Adjuvant.** To determine knee edema of the affected limb, the diameter across the arthritic knee joint was measured and compared to its contralateral knee joint at days 0, 14, 18 and 25 following initial Complete Freund’s Adjuvant (CFA) injection. **(A)** Docosahexaenoic acid (DHA) was given daily by oral administration (p.o.) from day 15 until day 25 after CFA injection. **(B)** Knee edema was significantly reduced at 10, 30 and 100 mg/kg doses of DHA in mice that received CFA injections compared to CFA-injected mice treated with vehicle. Each bar represents the mean ± SEM. *P < 0.05 vs. intraarticular (i.a.) CFA + p.o. vehicle by two-way repeated-measures analysis of variance followed by *post hoc* Bonferroni correction.

Repeated injections of CFA significantly increased knee edema in the ipsilateral knee joint as compared to the contralateral knee joint or in the knee joints of mice injected with saline solution (data not shown). In all mice that received CFA injections, knee edema was restricted to the ipsilateral joint with no visual evidence of knee edema in the contralateral hind limb, and this edema was maintained through day 25 after the initial CFA injection. At day 15 after the initial CFA injection, oral administration of DHA or vehicle was initiated and maintained until day 25. The anti-inflammatory effect of chronic oral administration of DHA at doses of 10, 30 and 100 mg/kg was determined at days 18 and 25 after the initial CFA injection (Figure 2A). The diameter of CFA-induced ipsilateral knee edema was reduced by DHA in a dose-dependent manner. This effect was statistically significant at doses of 10, 30 and 100 mg/kg (Figure 2B).

### Effect of acute oral administration of docosahexaenoic acid on pain-related behaviors and knee edema

**Figure 3:**
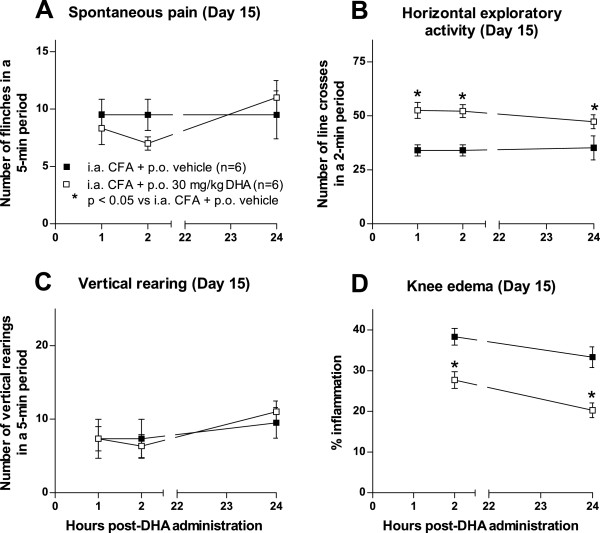
**Acute oral administration ofdocosahexaenoic acid restores horizontal exploratory activity and reduces knee edema.** At day 15, 30 mg/kg docosahexaenoic acid (DHA) was given orally (p.o.) to mice that received injections of Complete Freund’s Adjuvant (CFA). Behavioral analysis was performed at 1, 2 and 24 hours after DHA administration. Knee edema was measured at hours 2 and 24 after DHA administration. Acute oral administration of DHA did not modify either spontaneous pain **(A)** or vertical rearing **(C)** in CFA-injected mice. **(B)** Horizontal exploratory activity was increased after DHA administration as compared to CFA-injected mice treated with vehicle. **(D)** Knee edema was decreased at 2 and 24 hours after DHA administration. Each bar represents the mean ± SEM. *P < 0.05 vs. intraarticular (i.a.) CFA + p.o. vehicle according to respective times by two-way repeated-measures analysis of variance followed by *post hoc* Bonferroni correction.

To determine the effect of DHA in the CFA-induced arthritis model following a single administration, we performed behavioral analysis at day 15 after CFA injection. Behavioral analysis revealed that a single oral administration of DHA (30 mg/kg) did not significantly reduce the spontaneous nociceptive behaviors (Figure 3A) or restore the CFA-induced vertical rearing impairment at hours 1, 2 and 24 after DHA administration (Figure 3C). In contrast, acute DHA administration significantly reversed the CFA-induced horizontal exploratory activity impairment (Figure 3B) and reduced the knee edema of the ipsilateral knee joint at 2 and 24 hours as compared to CFA-injected mice treated with vehicle (Figure 3D).

### Effect of chronic intraarticular administration of docosahexaenoic acid on pain-related

**Figure 4:**
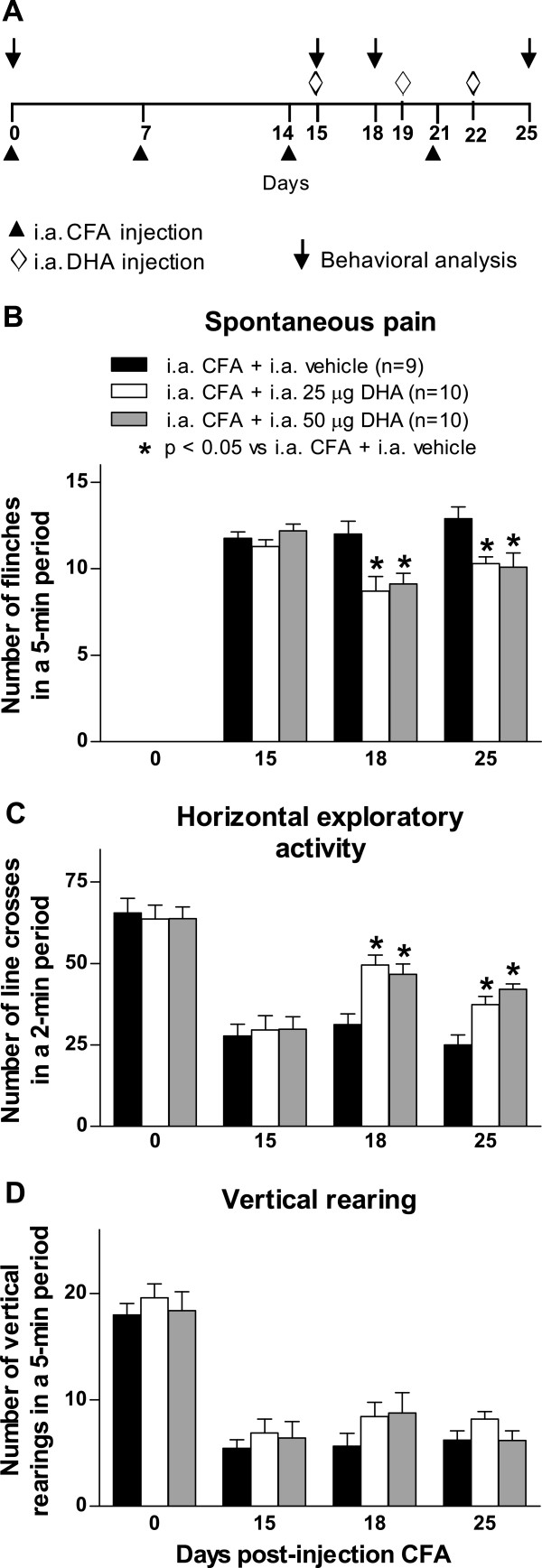
**Intraarticular administration of docosahexaenoic acid reduces pain-related behaviors in arthritis induced by Complete Freund’s Adjuvant.** Docosahexaenoic acid (DHA) or vehicle was intraarticularly (i.a.) administered at days 15, 19 and 22 after initial Complete Freund’s Adjuvant (CFA) injection into the right knee at doses of 25 μg/joint and 50 μg/joint. **(A)** Behavioral analysis was performed at days 0 and 15 (immediately prior to i.a. administration of DHA) and at days 19 and 25 following initial CFA injection. **(B)** The two doses of DHA significantly reduced the number of spontaneous flinches. **(C)** The horizontal exploratory activity increased significantly in CFA-injected mice following DHA treatment. **(D)** The i.a. administration of DHA did not significantly increase the number of vertical rearings as compared to CFA-injected mice treated with vehicle. Each bar represents the mean ± SEM. *P < 0.05 vs. i.a. CFA + i.a. vehicle according to its respective time by two-way repeated-measures analysis of variance followed by *post hoc* Bonferroni correction.

**Figure 5:**
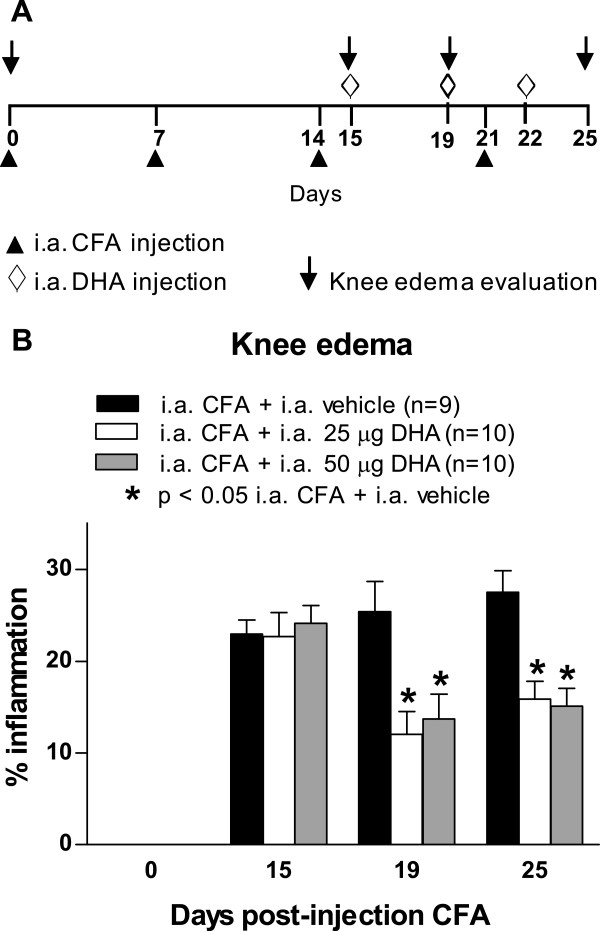
**Intraarticular administration of docosahexaenoic acid reduces knee edema in arthritis induced by Complete Freund’s Adjuvant.** To determine knee edema, the diameter across the arthritic knee joint was measured and compared to the contralateral knee joint at days 0, 15, 19 and 25 following initial Complete Freund’s Adjuvant (CFA) injection. **(A)** The intraarticular (i.a.) administration of docosahexaenoic acid (DHA) was performed at days 15, 19 and 22 using doses of 25 μg and 50 μg per mouse injected into the right knee. **(B)** The i.a. administration of DHA significantly reduced knee edema at doses of 25 μg/joint and 50 μg/joint at days 19 and 25 in CFA-injected mice compared to CFA-injected mice treated with vehicle. Each bar represents the mean ± SEM. *P < 0.05 vs. CFA + vehicle following two-way repeated-measures analysis of variance and *post hoc* Bonferroni correction.

To determine whether intraarticular administration of DHA would reduce pain-related behaviors and knee edema in the CFA-induce arthritis model, administration of DHA into the knee joint (25 and 50 μg/joint) was performed at days 15, 19 and 22 after initial CFA injection (Figure 4A). Both doses of DHA (25 and 50 μg/joint) decreased spontaneous pain behavior (Figure 4B). The CFA-induced impairment of horizontal exploratory activity was significantly restored by intraarticular DHA administration at both doses on day 25 (Figure 4C). The intraarticular DHA did not significantly change the CFA-induced reduction in vertical rearing (Figure 4D). The anti-inflammatory effect of chronic intraarticular administration of DHA at doses of 25 and 50 μg/joint was determined at days 18 and 25 after the initial CFA injection (Figure 5A). DHA treatment at doses of 25 and 50 μg/joint significantly decreased the diameter of the ipsilateral knee joint on the days evaluated (Figure 5B).

### Involvement of opioid mechanism in antinociceptive effect of docosahexaenoic acid

**Figure 6:**
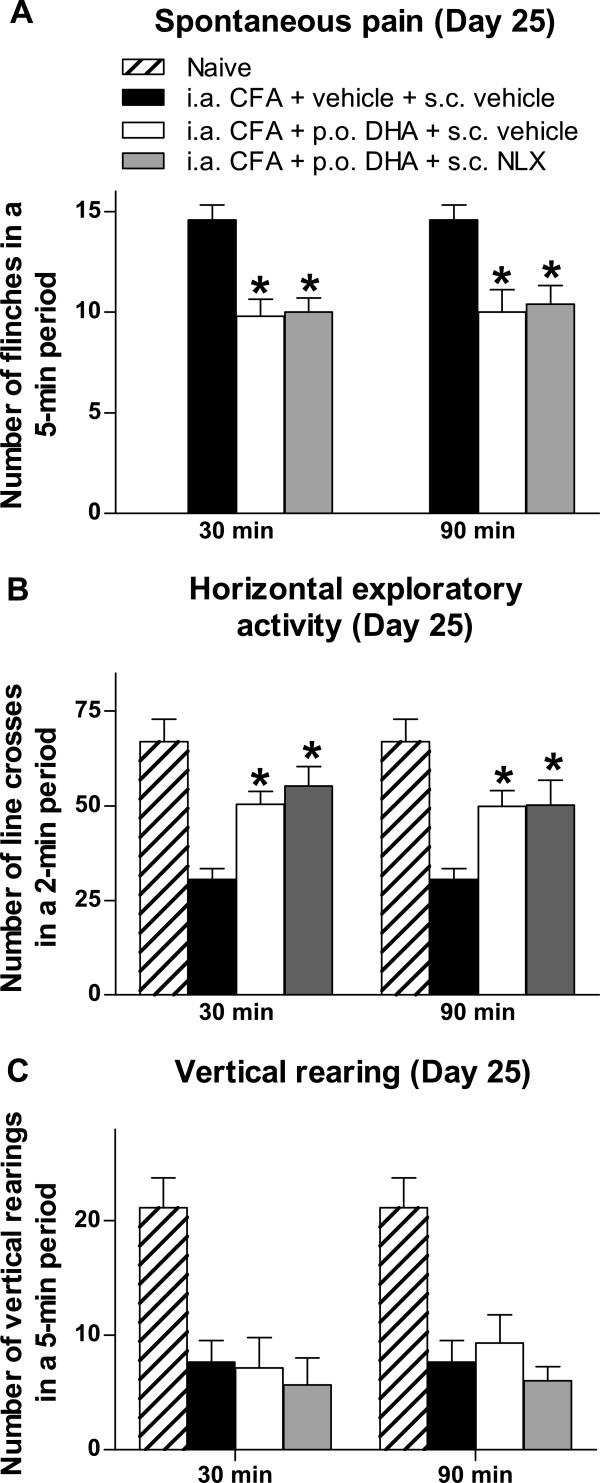
**Antinociceptive effect of docosahexaenoic acid was not attenuated by administration of naloxone in mice with CFA-induced arthritis.** Mice injected with Complete Freund’s Adjuvant (CFA) were treated daily with 30 mg/kg docosahexaenoic acid (DHA) or with vehicle from day 15 until day 25 after CFA injection. At day 25, 3 mg/kg naloxone (NLX) was administered subcutaneously (s.c.) 90 minutes after DHA administration. Behavioral analysis was performed at 30 minutes after NLX administration. The beneficial effect of DHA on spontaneous pain **(A)** and horizontal exploratory activity **(B)** was not significantly affected by NLX. The number of vertical rearings was not significantly affected by NLX **(C)**. i.a. = intraarticular; p.o. = oral. Each bar represents the mean ± SEM. *P < 0.05 vs. i.a. CFA + i.a. vehicle + s.c. vehicle by one-way analysis of variance followed by *post hoc* Bonferroni correction.

To evaluate a possible mechanism of action for the antinociceptive effect of DHA, we examined the effect of NLX, an opioid receptor antagonist, on DHA antinociceptive action in mice that received CFA injections and were treated with oral DHA. NLX did not reverse the antinociceptive effect of DHA on mice with CFA-induced arthritis (Figure 6). Additionally, NLX by itself did not increase or reduce CFA-induced spontaneous flinches or pain-related functional disabilities (data not shown).

## Discussion

Our present study shows an antinociceptive and anti-inflammatory effect of DHA following repeated systemic or intraarticular treatment in a mouse model of chronic CFA-induced knee arthritis. Although investigators in preclinical studies have reported that DHA has antinociceptive effects, traditional reflex tests of nociception were used in most of these studies (for example, hind paw withdrawal responses to thermal and mechanical stimuli) [32-34,36], and/or acute pain models have been utilized [32,33]. In our present study, we utilized a chronic pain model of knee arthritis to evaluate the effect of DHA on long-term measures of spontaneous nociception and pain-related functional deficits, as these measures more closely resemble painful conditions in clinical RA.

### Chronic administration of docosahexaenoic acid is more effective than acute administration in reducing pain-related behaviors

Although we observed that acute administration of DHA was able to significantly reduce knee edema and restore CFA-induced horizontal exploratory activity deficits, this treatment did not significantly reduce spontaneous flinching. Although we do not yet know the exact mechanisms underlying this finding, we suggest that CFA-induced arthritic pain might not be driven only by central and peripheral sensitization triggered by chronic inflammation of the knee [22]. In this context, several studies have demonstrated that osteoclast-mediated acidosis and bone loss are also involved in the generation and maintenance of arthritic pain [24,51,52]. Researchers in previous studies have shown that treatment with DHA decreases osteoclast activation [17,18] and that long-term intake of a mixture of ω-PUFAs (containing DHA) reduces urine excretion of markers of bone and cartilage resorption in patients with RA [53]. In light of these findings, we suggest that the greater beneficial effect of chronic DHA as compared to acute DHA on spontaneous pain and functional outcomes may be due to DHA’s ability to reduce arthritis-mediated bone loss and erosion, thereby preserving mechanical bone strength.

### Intraarticular administration is as effective as systemic administration at reducing pain and knee edema

Although RA is one of the most common musculoskeletal disorders in the world [1,4], current therapies used to alleviate joint pain have limited efficacy and/or are associated with significant side effects [9,11,54]. For example, owing to the chronic nature of the disease, systemic long-term treatment with nonsteroidal anti-inflammatory drugs and cyclooxygenase 2 (COX-2) inhibitors in patients with arthritic pain may induce negative side effects in the cardiovascular and gastrointestinal systems [8-11]. A therapeutic option frequently used to minimize systemic side effects is to deliver the drug directly into the affected joint (for example, by intraarticular administration) [28-31]. Although intraarticular administration has been widely used to deliver corticosteroids and other disease-modifying antirheumatic drugs [55,56], this method is not frequently used to deliver analgesics in arthritic conditions. In the present study, we evaluated the chronic effect of intraarticular administration of DHA on spontaneous nociception, knee edema and pain-related functional disabilities. Our results show that intraarticular injection of DHA was as effective as oral administration. However, it should be noted that for intraarticular administration, we used doses 80 times lower than the systemic concentration. Given that intraarticular injections may be associated with discomfort, pain and possible risk of infection [31,57], the number of intraarticular injections should be kept to a minimum. In this study, DHA intraarticular injections were performed twice per week versus on a daily basis for oral administration; however an analgesic and anti-inflammatory effect of DHA was reached. Thus, the results of this study suggest that local delivery of DHA into the affected joints may offer another alternative treatment for arthritic pain and inflammation without the possible development of side effects associated with systemic administration.

### Antinociceptive effect of docosahexaenoic acid on arthritic pain does not appear to involve an endogenous opioid mechanism

Investigators in previous studies have demonstrated that pretreatment with NLX prevented the antinociceptive effect of DHA after oral administration in acute pain models including formalin-induced nociception and acetic acid writhing tests in the mouse [32]. Additionally, oral administration of DHA increases plasma levels of β-endorphins in mice [58]. These data, taken together, suggest that DHA exerts an antinociceptive effect via activation of an endogenous opioidergic mechanism. In contrast, our experiments demonstrated that administration of NLX (using a dosage and scheme of administration in mice previously validated by others and in our laboratory) [49,50] did not reverse the beneficial effect of DHA on CFA-induced flinching and horizontal activity deficit. The reasons for these findings are unclear; however, it is possible that mechanisms underlying DHA antinociception in acute pain models may be different from those involved in chronic pain states and/or there may be a decrease in the levels of endorphins in chronic arthritic conditions. In support of this theory, investigators in a clinical study demonstrated that there is an inverse correlation between serum levels of β-endorphins and rheumatoid disease activity score and the duration of RA [59].

### Possible mechanisms underlying anti-inflammatory and antinociceptive actions of docosahexaenoic acid

Although researchers in several studies have demonstrated anti-inflammatory and antinociceptive effects of DHA following acute administration [32,34,36,60], less is known about the molecular mechanisms underlying these effects. Our possible hypothesis, gleaned from basic scientific literature, is that both direct and indirect effects may explain the biological actions of DHA. First, direct effects may include competition of DHA with arachidonic acid as a substrate for cyclo- and lipoxygenase, thus reducing the production of inflammatory eicosanoids [26,61,62]. Moreover, researchers recently demonstrated that the anti-inflammatory actions of DHA may partially be explained by inhibition of lipopolysaccharide-mediated COX-2 induction and activity via direct activation of the free fatty acid receptor 4 [63]. However, indirect effects may include the participation of D-resolvins and protectin D1, which are lipid mediators enzymatically synthesized *in vivo* from DHA and promote the resolution of inflammation with greater potency than their parent precursors [35,64].

The mechanisms underlying the antinociceptive actions of DHA in arthritic pain conditions remain to be explored. However, the results of a previous study suggest that the anti-inflammatory actions of DHA may also contribute to amelioration of arthritic pain [26]. It also has been shown that DHA may directly modulate the excitability of sensory neurons through inhibition of the voltage-dependent sodium currents in dorsal root ganglion neurons [65]. However, an indirect analgesic effect of DHA on arthritic conditions cannot be ruled out. It was recently shown that DHA affects disease progression in that it reduces knee synovial inflammation, pannus formation, cartilage damage and bone damage as evaluated by histopathological analysis in a collagen-induced arthritis model in the mouse [26]. Both direct actions on sensory neurons and effects on disease progression, in addition to as yet unknown mechanisms, presumably participate in the analgesic actions of DHA in arthritic conditions.

The present study has some limitations. First, we suggest that the beneficial effect of DHA on pain-related functional disabilities may partially be a result of DHA’s inhibiting osteoclast-mediated bone destruction and preserving bone mechanical strength. Whether DHA affects bone mass and microarchitecture in arthritic conditions is unknown. Second, it is possible that intraarticular delivery of olive oil by itself could further increase pain, discomfort and synovial inflammation. However, our results show that treatment-naïve animals that received multiple intraarticular olive oil injections displayed minimal spontaneous flinching and that levels of locomotor activity and vertical rearing were similar in magnitude as those of treatment-naïve mice. The mechanisms driving anti-inflammatory and antinociceptive actions in arthritic conditions by DHA remain to be elucidated. Although our present study suggests that an endogenous opioid pathway does not seem to be involved in the actions of DHA, future studies need to be performed to explore the molecular targets of DHA in painful arthritic conditions.

## Conclusions

In our present study, we show that either oral or intraarticular administration of DHA significantly reduced spontaneous flinching behavior and knee edema and improved horizontal exploratory activity in a unilateral CFA-induced knee arthritis model. These results suggest that DHA may be a candidate for further testing as a therapeutic alternative in the treatment of pain-related functional disabilities in patients with RA.

## Abbreviations

CFA: Complete Freund’s adjuvant; DHA: Docosahexaenoic acid; EPA: Eicosapentaenoic acid; RA: Rheumatoid arthritis; ω-3 PUFA: Ω-3 polyunsaturated fatty acid.

## Competing interests

The authors declare that they have no competing interests.

## Authors’ contributions

AMTG designed and executed the experiments and drafted the manuscript. CEMU and PAAV designed and executed the experiments and revised the manuscript. RIAG analyzed and interpreted the data and revised the manuscript. AECP performed data analysis and wrote and critically revised the manuscript. RMMR analyzed and interpreted the data and wrote the manuscript. JMJA conceived of and designed the study, analyzed the data and wrote the manuscript. All authors read and approved the final manuscript.
